# Splenosis: A Rare Etiology for Bowel Obstruction—A Case Report and Review of the Literature

**DOI:** 10.1155/2015/890602

**Published:** 2015-10-12

**Authors:** George Younan, Edward Wills, Gordon Hafner

**Affiliations:** ^1^Division of Surgical Oncology, Department of Surgery, Medical College of Wisconsin, 9200 W. Wisconsin Avenue, Milwaukee, WI 53226, USA; ^2^Department of Surgery, Inova Fairfax Hospital, 3300 Gallows Road, Falls Church, VA 22041, USA

## Abstract

Splenosis is a historically uncommon etiology for bowel obstruction. Autotransplanted splenic tissues following surgery or trauma of the spleen are known to occur in multiple locations of the abdominal cavity and pelvis. The small bowel mesentery is a blood vessel-rich environment for growth of splenic fragments. We present a case of a 36-year-old male patient who sustained a gunshot wound to his left abdomen requiring a splenectomy and bowel resection fifteen years prior to his presentation with small bowel obstruction requiring exploration, adhesiolysis, and resection of the mesenteric splenic deposit. Our aim in this report is to provide awareness of splenosis as an etiology for bowel obstruction, especially with increased incidence and survival following abdominal traumas requiring splenectomies. We also stress on the importance of history and physical examination to include splenosis on the list of differential diagnoses for bowel obstruction.

## 1. Introduction

Splenic fragments after trauma or surgery have the ability to heterotopically autotransplant onto vascularized intra- or extraperitoneal surfaces [1, 2]. Most common locations of splenic deposits are in the vicinity of the left upper quadrant, typically involving the serosal surfaces of small and large bowel, the greater omentum, and diaphragm, but sometimes they can grow in the nearby mesentery or be found in very unusual locations [3–5]. Most cases are asymptomatic and are incidentally diagnosed during imaging studies or surgical explorations for other conditions [1, 6]. We present in this report a case of mesenteric splenosis resulting in adhesive small obstruction requiring exploration, adhesiolysis, and splenosis resection.

## 2. Case Presentation

**Figure 1:**
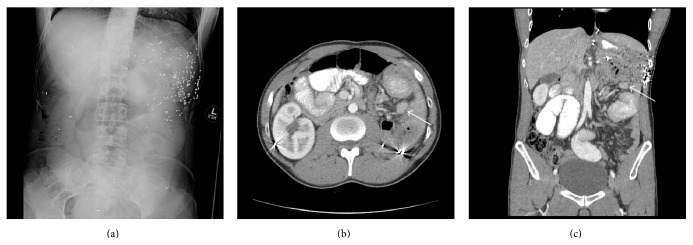
Abdominal X-ray shown in (a), depicting the number of shrapnel left in the left side of the body after the gunshot injury, in addition to dilated loops of bowel. CT scan with oral and intravenous contrast shows the splenosis deposit (white arrow) in axial (b) and coronal (c) views.

**Figure 2:**
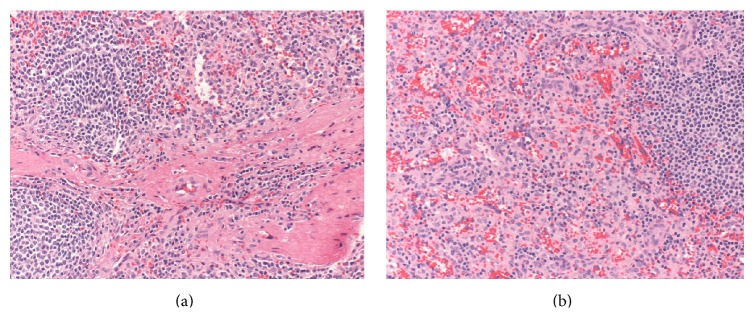
H&E stained slides of the splenosis deposit showing splenosis architecture. Splenic nodules separated by strands of fibrous trabeculae (a). White and red pulp nodules are seen (b).

A 36-year-old man with a history of a gunshot trauma to the left upper quadrant at the age of thirty-one, requiring multiple operations, including a splenectomy, resulting in recurrent small bowel obstructions over fifteen years. While previous episodes of obstruction resolved with nonoperative management, his recent one required surgical exploration. A preoperative abdominal film showed the remaining gunshot shrapnel (Figure 1(a)), and a preoperative CT scan showed a transition point in the left upper quadrant and a small 2 × 3 cm hyperintense mass (Figures 1(b) and 1(c)). Upon exploration and adhesiolysis, a well-contoured, dark blue mass was found in the small bowel mesentery at the transition point, correlating to the CT scan findings and causing scarring and shortening of the mesentery resulting in a mechanical small bowel obstruction. The mass was resected off the mesentery and sent for pathology which showed that it consisted with a splenosis deposit with architecture of nodules of splenic tissue, surrounded by thick connective tissue trabeculae (Figure 2). The patient recovered and was discharged home on a regular diet.

## 3. Discussion

Ectopic splenic tissues are classified into two types, congenital and acquired. Failure of union of the normal spleen during embryogenesis results in formation of accessory spleens. These are common and found in 20% of people [7]. They have a normal splenic histological architecture and function and commonly are found in the splenic hilum and the gastrosplenic, splenorenal, or splenocolic ligaments and they get their blood supply from a branch of the splenic artery [7, 8]. Splenosis is an acquired form of ectopic splenic tissues that gained function and perfusion after autotransplantation onto blood vessel-rich intra- or extraperitoneal environments; as a result their blood vessels are not related to the splenic artery [1, 9]. The mechanism of spread of splenic fragments is believed to be either by a direct seeding process onto adjacent surfaces or by hematological spread to distant organs such as the liver, breast, and brain [10, 11]. In support of the seeding process hypothesis, splenosis deposits are found in the chest in cases of traumatic diaphragmatic rupture exclusively in the left hemithorax, in subcutaneous locations where port sites were introduced, and in exit wounds after gunshot wounds that involved the spleen [1, 7, 12, 13].

The first report of a splenosis case dates back to 1896; it was described by Albrecht in Germany [14]. Buchbinder and Lipkoff in 1939 were the first to use the term “splenosis” [15]. The majority of splenosis cases occur after traumatic splenectomies, whereas a few are the result of elective splenectomies done for hematological diseases [1, 4]. A few reviews cite that traumatic splenosis is responsible for up to 93% of all splenosis cases [1, 12, 16]. There are reports that splenosis occurs in 16%–67% of patients after splenectomies for trauma or hematological diseases [11, 16–18]. The mean interval between a splenectomy and the clinical diagnosis of splenosis varies from a few months up to four decades [1, 12].

Most of the splenoses are asymptomatic and deposits are found incidentally on imaging studies or explorations done for other pathologies [19]. Ectopic splenic tissues after a splenectomy are either beneficial or harmful depending on the etiology leading to the splenectomy. Whereas splenic tissues provide some salvage splenic function after traumatic splenectomies, they usually are functional enough to cause recurrence of hematological diseases that the original splenectomies were meant to treat [16, 20, 21]. In addition, the risk of overwhelming postsplenectomy infection appears to be lower in trauma cases compared to elective splenectomies [21]. Whether the actual splenosis deposits confer such benefit and whether the matter of autologic splenic transplantation after a traumatic splenectomy would be beneficial are still a matter of debate [27]. Splenosis tissues cause one of three surgically significant conditions: intra- or extraluminal bleeding, local compression of adjacent tissues and organs, and misdiagnoses worrisome for tumors leading to unnecessary surgical explorations. Accessory spleens however rarely cause these conditions [28, 29].

Diagnosis of splenosis, as mentioned above, is in general confirmed after imaging studies and surgical explorations for other conditions. In our patient, it was diagnosed after exploration for adhesiolysis and relief of the small bowel obstruction, which should be and was done irrespective of the splenosis diagnosis. However a high level of suspicion should be exercised in cases where an unnecessary procedure can be avoided, especially in patients with a history of splenectomy [30]. In addition to a proper history and physical exam, a blood smear showing the absence of Howell-Jolly, Pappenheimer, or Heinz bodies is indicative of the presence of functional splenic tissues. However, at this day and age, imaging studies evolved to accurately diagnose splenosis deposits. Standard CT and MRI scans are able to identify and describe the anatomical locations of these deposits with a reasonable sensitivity [19]. Ferumoxide-enhanced MRI has been recently used for a better sensitivity in detecting iron oxide particles that are taken up by the reticuloendothelial system of the spleen and liver [31, 32]. Technetium (Tc) radionuclide scanning is considered the gold standard for the diagnosis of splenosis. Tc 99m tagged heat-damaged autologous red blood cells or indium 111-labeled platelets scintigraphy is more sensitive whenever liver and spleen tissues need to be differentiated [33, 34].

We describe in Table 1 all the reported cases in the literature where a splenosis was the cause of bowel obstruction and whether or not exploration is warranted.

In conclusion, splenosis is a benign condition involving autotransplantation of splenic tissues onto different surface or distant organs on the abdominal cavity. Although mostly asymptomatic, splenosis deposits have the ability to cause multiple morbidities to the patients, ranging between pain, bleeding, and obstruction, subjecting them to unnecessary surgeries. High clinical suspicion should be exercised in splenectomized patients to avoid invasive procedures, given that asymptomatic splenosis deposits pose no risk and can be left unresected.

## Figures and Tables

**Table 1:** 

Author	Journal	Year	Type of obstruction	Procedure
Obokhare et al. [6]	Journal of Gastrointestinal Surgery	2012	Colon	Left hemicolectomy, appendectomy

Gincu et al. [22]	Endoscopy	2011	Colon	Resection of mass

Garaci et al. [23]	World Journal of Gastroenterology	2009	Small bowel	Lysis of adhesions, resection of rectal mass

Sato et al. [24]	Pediatric Surgery International	2007	Small bowel	Laparotomy, lysis of adhesions

Abeles and Bego [25]	Surgical Endoscopy	2003	Small bowel (intussusception)	Laparoscopic-assisted small bowel resection

Sirinek et al. [26]	Southern Medical Journal	1984	Small bowel	Laparotomy, lysis of adhesions

## References

[1] Ksiadzyna D., Peña A. S. (2011). Abdominal splenosis. *Revista Espanola de Enfermedades Digestivas*.

[2] Akay S., Ilica A. T., Battal B., Karaman B., Guvenc I. (2012). Pararectal mass: An atypical location of splenosis. *Journal of Clinical Ultrasound*.

[3] Sikov W. M., Schiffman F. J., Weaver M., Dyckman J., Shulman R., Torgan P. (2000). Splenosis presenting as occult gastrointestinal bleeding. *American Journal of Hematology*.

[4] Brewster D. C. (1973). Splenosis. Report of two cases and review of the literature. *The American Journal of Surgery*.

[5] Papakonstantinou E., Kalles V., Papapanagiotou I. (2013). Subcutaneous splenosis of the abdominal wall: report of a case and review of the literature. *Case Reports in Surgery*.

[6] Obokhare I. D., Beckman E., Beck D. E., Whitlow C. B., Margolin D. A. (2012). Intramural colonic splenosis: a rare case of lower gastrointestinal bleeding. *Journal of Gastrointestinal Surgery*.

[7] Moon C., Choi Y.-J., Kim E. Y. (2013). Combined intrathoracic and intraperitoneal splenosis after splenic injury: case report and review of the literature. *Tuberculosis and Respiratory Diseases*.

[8] Mortele K. J., Mortele B., Silverman S. G. (2004). CT features of the accessory spleen. *American Journal of Roentgenology*.

[9] Pearson H. A., Johnston D., Smith K. A., Touloukian R. J. (1978). The born-again spleen. Return of splenic function after splenectomy for trauma. *The New England Journal of Medicine*.

[10] Kwok C.-M., Chen Y.-T., Lin H.-T., Su C.-H., Liu Y.-S., Chiu Y.-C. (2006). Portal vein entrance of splenic erythrocytic progenitor cells and local hypoxia of liver, two events cause intrahepatic splenosis. *Medical Hypotheses*.

[11] di Costanzo G. G., Picciotto F. P., Marsilia G. M., Ascione A. (2004). Hepatic splenosis misinterpreted as hepatocellular carcinoma in cirrhotic patients referred for liver transplantation: report of two cases. *Liver Transplantation*.

[12] Losanoff J. E., Jones J. W. (2001). Splenosis after laparoscopic splenectomy. *Surgical Endoscopy*.

[13] Yeh C.-J., Chuang W.-Y., Kuo T.-T. (2006). Unusual subcutaneous splenosis occurring in a gunshot wound scar: pathology and immunohistochemical identification. *Pathology International*.

[14] Cohen E. A. (1954). Splenosis: review and report of subcutaneous splenic implant. *A.M.A. Archives of Surgery*.

[15] Buchbinder J. H., Lipkoff C. J. (1939). Splenosis: multiple peritoneal splenic implants following abdominal injury. *Surgery*.

[16] Mazur E. M., Field W. W., Cahow C. E., Schiffman F. J., Duffy T. P., Forget B. G. (1978). Idiopathic thrombocytopenic purpura occurring in a subject previously splenectomized for traumatic splenic rupture. Role of splenosis in the pathogenesis of thrombocytopenia. *The American Journal of Medicine*.

[17] Müller U., Röthlin M. (1995). Splenic neoformation following trauma-induced splenectomy—diagnosis and function. *Swiss Surgery*.

[18] Livingston C. D., Levine B. A., Lecklitner M. L., Sirinek K. R. (1983). Incidence and function of residual splenic tissue following splenectomy for trauma in adults. *Archives of Surgery*.

[19] Lake S. T., Johnson P. T., Kawamoto S., Hruban R. H., Fishman E. K. (2012). CT of splenosis: patterns and pitfalls. *American Journal of Roentgenology*.

[20] Corazza G. R., Tarozzi C., Vaira D., Frisoni M., Gasbarrini G. (1984). Return of splenic function after splenectomy: how much tissue is needed?. *British Medical Journal*.

[21] Khosravi M. R., Margulies D. R., Alsabeh R., Nissen N., Phillips E. H., Morgenstern L. (2004). Consider the diagnosis of splenosis for soft tissue masses long after any splenic injury. *American Surgeon*.

[22] Gincu V., Kornprat P., Thimary F., Jahn S., Mischinger H. J. (2011). Intestinal obstruction caused by splenosis at the rectosigmoid junction, mimicking malignant pelvic tumor. *Endoscopy*.

[23] Garaci F. G., Grande M., Villa M. (2009). What is a reliable CT scan for diagnosing splenosis under emergency conditions?. *World Journal of Gastroenterology*.

[24] Sato M., Motohiro T., Seto S., Kogire M., Takada K., Hamada Y. (2007). A case of splenosis after laparoscopic splenectomy. *Pediatric Surgery International*.

[25] Abeles D. B., Bego D. G. (2003). Occult gastrointestinal bleeding and abdominal pain due to entero-enteric intussusception caused by splenosis. *Surgical Endoscopy and Other Interventional Techniques*.

[26] Sirinek K. R., Livingston C. D., Bova J. G., Levine B. A. (1984). Bowel obstruction due to infarcted splenosis. *Southern Medical Journal*.

[27] Pisters P. W. T., Pachter H. L. (1994). Autologous splenic transplantation for splenic trauma. *Annals of Surgery*.

[28] Conway A. B., Cook S. M., Samad A., Attam R., Pambuccian S. E. (2013). Large platelet aggregates in endoscopic ultrasound-guided fine-needle aspiration of the pancreas and peripancreatic region: a clue for the diagnosis of intrapancreatic or accessory spleen. *Diagnostic Cytopathology*.

[29] Yu H., Li T., Xia L. (2009). Intrahepatic splenosis mimicking hepatoma. *BMJ Case Reports*.

[30] Kang K. C., Cho G. S., Chung G. A. (2011). Intrahepatic splenosis mimicking liver metastasis in a patient with gastric cancer. *Journal of Gastric Cancer*.

[31] Berman A. J., Zahalsky M. P., Okon S. A., Wagner J. R. (2003). Distinguishing splenosis from renal masses using ferumoxide-enhanced magnetic resonance imaging. *Urology*.

[32] Prosch H., Oschatz E., Pertusini E., Mostbeck G. (2006). Diagnosis of thoracic splenosis by ferumoxides-enhanced magnetic resonance imaging. *Journal of Thoracic Imaging*.

[33] Armas R. R. (1985). Clinical studies with spleen-specific radiolabeled agents. *Seminars in Nuclear Medicine*.

[34] Schiff R. G., Leonidas J., Shende A., Lanzkowski P. (1987). The noninvasive diagnosis of intrathoracic splenosis using technetium-99m heat-damaged red blood cells. *Clinical Nuclear Medicine*.

